# T cells in abdominal aortic aneurysm: immunomodulation and clinical application

**DOI:** 10.3389/fimmu.2023.1240132

**Published:** 2023-08-18

**Authors:** Wei Gong, Yu Tian, Lei Li

**Affiliations:** Department of Vascular Surgery, The Second Affiliated Hospital of Dalian Medical University, Dalian, Liaoning, China

**Keywords:** T cells, abdominal aortic aneurysm, immunomodulation, immunotherapy, clinical application

## Abstract

Abdominal aortic aneurysm (AAA) is characterized by inflammatory cell infiltration, extracellular matrix (ECM) degradation, and vascular smooth muscle cell (SMC) dysfunction. The inflammatory cells involved in AAA mainly include immune cells including macrophages, neutrophils, T-lymphocytes and B lymphocytes and endothelial cells. As the blood vessel wall expands, more and more lymphocytes infiltrate into the outer membrane. It was found that more than 50% of lymphocytes in AAA tissues were CD3^+^ T cells, including CD4^+^, CD8^+^T cells, γδ T cells and regulatory T cells (Tregs). Due to the important role of T cells in inflammatory response, an increasing number of researchers have paid attention to the role of T cells in AAA and dug into the relevant mechanism. Therefore, this paper focuses on reviewing the immunoregulatory role of T cells in AAA and their role in immunotherapy, seeking potential targets for immunotherapy and putting forward future research directions.

## 1 Introduction

**Figure 1:**
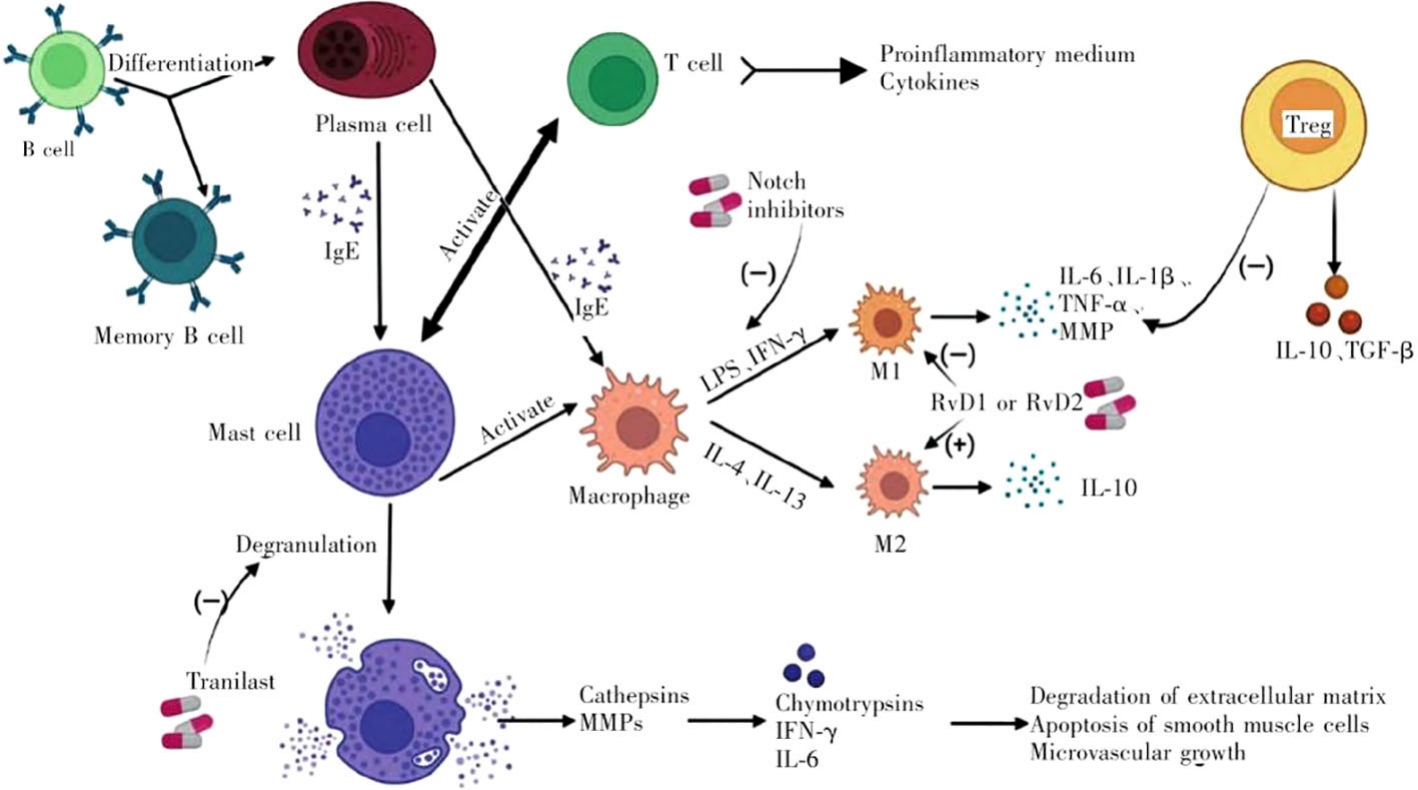
Immune cells involved in the progression of AAA and their interactions. (LPS, lipopolysaccharide; IFN-γ, interferon-γ; IL, interleukin; TGF-β, transforming growth factor-β; TNF-α, tumor necrosis factor-α; MMP, matrix metalloproteinase; Treg, regulatory T cell).

Abdominal aortic aneurysm (AAA) is defined as a aneurysm with permanent aneurysm that is more than 50% larger than normal or larger than 3 cm in diameter (1). Epidemiological studies show that with the aging of the population, the smoking population increases, and the incidence of AAA increases year by year. The main treatment for AAA is surgical repair, but more than 90% of aneurysms are small aneurysms (3.0cm < diameter < 5.5cm) (2). For these patients, surgical treatment is not appropriate, so drug therapy has aroused great concern. Unfortunately, the drug options so far have been very limited, and further exploration of the underlying mechanisms of AAA is urgent (3). AAA is characterized by inflammatory cell infiltration, extracellular matrix degradation, and SMC dysfunction, which are associated with the infiltration of inflammatory cells in the outer and inner membranes of blood vessels. These factors together promote vascular remodeling and the weakening of the aortic wall (4). Vascular inflammation in AAA involves chemotaxis of inflammatory cells and release of pro-inflammatory factors, thus initiating a series of inflammatory responses (5). The inflammatory cells involved in AAA mainly include immune cells including macrophages, neutrophils, mast cells, natural killer (NK) cells, dendritic cells (DCs), B cells and T cells (6). T cells play a prominent role, in which CD4+T helper cell plays a leading role (7). Th1 cells typically produce interferon-gamma (IFN-γ), IL-2, and tumor necrosis factor (TNF), while Th2 cells secrete IL-4, IL-5, IL-10, and IL-13. Th1 cytokines frequently cause cellular inflammatory responses, such as activation of macrophages. Activated macrophages infiltrate aortic tissue and secrete extracellular matrix degradation such as matrix metalloproteinase-1 (MMP-1), 2 (MMP-2) and 9 (MMP-9), which directly contribute to AAA formation (8). The M1 and M2 macrophage phenotypes also have a significant impact on AAA. M2 macrophages are anti-inflammatory, whereas M1 macrophages exhibit pro-inflammatory characteristics. Interventions that prevent M2 from transiting to M1 or promote the transformation of macrophages into M2 may be of great help to AAA treatment. CD4 (+) CD25 (+) Treg cells play a key role in the transformation of macrophages into M2 (9). IL-4 and IL-13 can enhance the proliferation of B cells and activate Mast cell. AAA usually shows local deposition of immunoglobulin (10), which reflects that humoral immunity may participate in the pathogenesis of AAA, and promote the formation of AAA by secreting Collagenase and Elastase (11). The activated Mast cells release their granular contents, such as histamine, protein Hydrolase and inflammatory cytokines (IFN-γ, IL-6) is involved in the degradation of extracellular matrix, apoptosis of smooth muscle cells and angiogenesiss (12). (**Figure 1**) Among them, T cells play a prominent role.

Due to the important role of T cells in inflammatory response, an increasing number of researchers have paid attention to the role of T cells in AAA and dug into the relevant mechanism. Therefore, this paper focuses on reviewing the immunoregulatory role of T cells in AAA and their role in immunotherapy, seeking potential targets for immunotherapy and putting forward future research directions.

## 2 Immunomodulation of T cells in AAA

As the blood vessel wall expands, more and more lymphocytes infiltrate into the outer membrane. It was found that more than 50% of lymphocytes in AAA tissues were CD3+ T cells, including CD4^+^, CD8^+^T cells, γδ T cells and Tregs (13). Based on their immunophenotypes, T cells can be split into a number of subsets, primarily cytotoxic CD8^+^ T cells and CD4^+^ T helper cells. CD4^+^ T cells can be further divided into Th1(T-bet), Th2(GATA3), Th9(PU.1 IRF4), Th17(RORt), Th22(AhR RORγt), follicular helper T cells (Tfh), and Tregs (CD4^+^ CD25^+^ FoxP3^+^), each of which produce specific effector cytokines under unique transcriptional regulation (14). While CD4^+^ helper T cells are generally identical in terms of shape and cell membrane structure, their unique cytokine profiles, transcription factor expression patterns, and functional roles in immune responses distinguish them from each other (15). Different T helper cell (Th cells) subsets have distinctive cytokine production patterns. The main source of IFN-γ, which supports cell-mediated immune responses, is TH1 cells. Contrarily, TH2 cells generate cytokines such interleukin-4 (IL-4), interleukin-5 (IL-5), and interleukin-13 (IL-13), which are essential for allergic reactions and anti-parasite immunity. TH17 cells release the inflammatory mediators interleukin-17 (IL-17) and interleukin-22 (IL-22), which aid in the immune system’s defense against fungus (14). On the other hand, by the release of transforming growth factor-beta (TGF-β) and interleukin-10 (IL-10). Treg cells play a critical role in immune response control and maintenance of immunological tolerance (16). CD8^+^ T cells are a type of T lymphocyte that expresses the CD8 protein on their cell surface. The main function of CD8^+^ T cells is cytotoxicity, also known as cell killing. When they encounter cells infected with viruses or other intracellular pathogens, CD8^+^ T cells can recognize and bind to specific antigens presented by infected cells (17). This triggers the activation of CD8^+^ T cells and leads to the release of cytotoxic molecules, such as perforin and granzymes, which induce apoptosis (cell death) in the infected cells (18). Unlike conventional CD4^+^ or CD8^+^ T cells that use alpha beta (αβ) TCR, γδT cells possess a TCR comprised of gamma and delta chains. They can detect a variety of antigens without the aid of molecules from the major histocompatibility complex (MHC), thanks to their own TCR (19).

This section will discuss the roles and specific mechanisms of different T cell subsets in the formation and progression of AAA.

### 2.1 CD4^+^ T cells

#### 2.1.1 T helper cells

Xiong et al. found that CaCl_2_ could not induce AAA formation in mice with CD4^+^ T cell defects, suggesting that CD4^+^ T cells play an integral role in the progression of AAA (20). However, the regulation mechanism of Th cells subgroup 1 (Th1),2 (Th2) and 17 (Th17) and their secreted cytokines involved in AAA is very complex. The most common CD4^+^ T cell in human AAA tissue is Th2, while Th1 cells are rare, so Th2 cells are believed to play a dominant role in the progression of human AAA (21). Th2 cells are characterized by the production of type 2 cytokines such as IL-4, IL-5, IL-9 and IL-10. Atherosclerotic lesions have been demonstrated to be impacted by these cytokines. Some research indicates that the use of rIL-9 market increased the plaque area, which was connected to a rise in VCAM-1 expression and a propensity for macrophage and T cell infiltration. Downregulation of IL-9 by anti-IL-9 mAb and LncRNA CASC11 induced the opposite effect (22, 23) and research by Brown and colleagues suggests that IL-9 reducing smooth muscle 22α and may promote phenotype transformation of SMC through the STAT3 pathway, which may exacerbate vascular dysfunction and lead to the formation of AAA (24). The development of AAA is slowed down by IL-10 (25).The vulnerability of IL-10(-/-) mice to Ang II-induced AAA and aortic rupture was observed to be enhanced (26). However, a lack of IL-4 just minimally changes how atherosclerosis develops (27, 28), IL-5 deficiency, however, has been proven to hasten atherosclerosis (28). Notably, IL-4 and IL-5 produced by invading Th2 cells in AAA tissue were thought to be harmful, especially because they can cause vascular smooth muscle cells to undergo apoptosis (29, 30). Additionally, Shimizu et al. showed that Th1/Th2 cytokine balance plays an important role in regulating matrix remodeling and is significant in the pathophysiology of aneurysms and atherosclerosis, indicating that the transformation of Th1 cells to Th2 cells is related to AAA augmentation (31). However, IL-4 and IL-5 may also be secreted by ILC2 and NK cells and perform similar functions to Th2 cells (32). Because NK cells and ILC2 also release type II cytokines, it is difficult to say if the unique role of Th2 cells in AAA is connected to this. Type 2 cytokine sources unique to particular cells have not been thoroughly investigated in AAA, so it will be crucial for future investigations to address cell specificity (33, 34).

**Figure 2:**
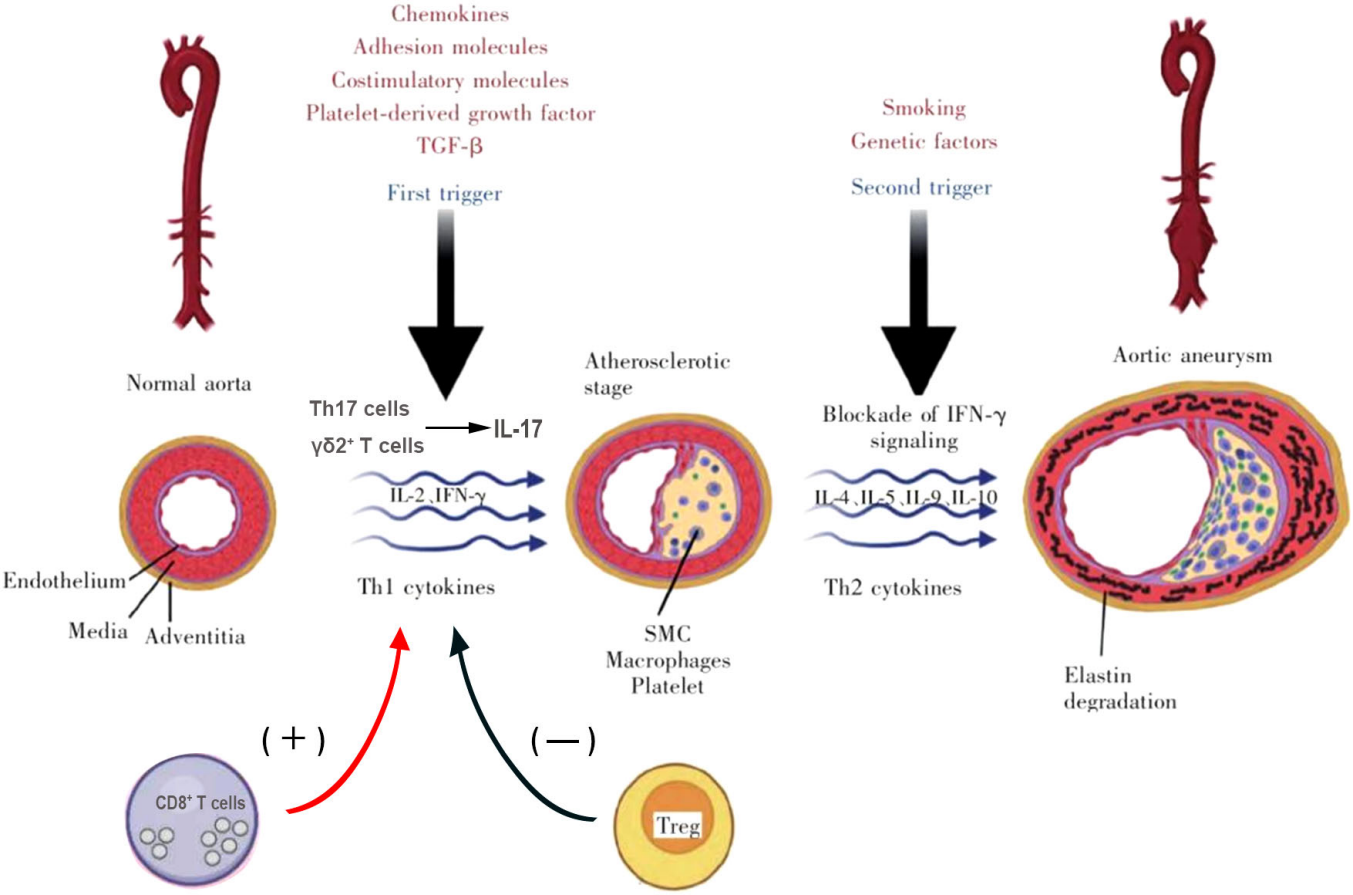
Mechanism of action of T cells in AAA formation.

Th1 cells have been shown to play a pro-inflammatory role in atherosclerosis mainly through the production of IFN-γ (35, 36). According to earlier research, intraperitoneal IFN-γ may partially restore AAA in CD4(-/-) mice. Furthermore, MMP production is reduced and AAA development is inhibited in mice with a targeted ablation of IFN-γ (20). Confusingly, King et al.’s angiotensin II-induced mice model found that IFN-γ insufficiency was linked to AAA enhancement (37), suggesting that this cytokine plays a protective effect in AAA. In particular, IFN-γ regulates the production of CXCL10 in AAA, which in turn regulates the attraction of protective T cells (37). These two contrasting results make the role of IFN-γ in AAA unclear. Further studies are needed to investigate its role in different stages of the disease, its induction mechanism, cell-specific IFN-γR signaling, and cell-type-specific mechanisms of IFN-γ production. In addition, Zhang et al. (38). used recombinant leptin to intervene in angiotensin II-induced AAA mouse model and found that leptin attenuates AAA formation. According to the study, leptin increases T-bet, a crucial transcription factor for Th1 polarization (38), suggesting that intervention of T-bet expression can also regulate the progression of AAA. Another proinflammatory cytokine released by Th1 cells called IL-2 is capable of accelerating atherosclerosis. According to prior research, intraperitoneal IL-2 injection into ApoE/mice fed an HF diet increases atherosclerosis while anti-IL-2 antibody treatment has a protective effect (39). However, IL-2 appears to be advantageous in AAA. Foxp3+Treg is produced by IL-2 complex therapy, which also slows the growth of angiotensin II-driven AAA and lowers mortality in ApoE -/- mice (40). The mechanism of action of Th1 and Th2 cells is shown in **Figure 2**.

The Th17 subgroup is also involved in immune regulation of AAA progression (13) and is regulated by the transcription factor RORγt, characterized by the production of cytokine IL-17A, IL-17F, and IL-22 (41). Th17 cells primarily have an immune-stimulating and pro-inflammatory role, which accelerates the development of several inflammatory illnesses, including atherosclerosis (42–44). In the elastase perfusion model of AAA constructed by Sharma et al., CD4^+^ T cell-produced IL-17 promoted the occurrence of inflammation, thus inducing the formation of AAA, while the absence of IL-17 limited the progression of the disease (45). The considerable attenuation of TNF, IFN, and MCP-1 in elastase-perfused IL-17/IL-17 mice aortas were of special interest.These cytokines are elevated in human AAA and have been proven in animal studies to induce aneurysm development (20, 46, 47). The fact that IL-17 controls the production of these inflammatory cytokines shows that IL-17 produced by CD4+ T cells is a key player in the early stages of the AAA-related inflammatory cascade. Similarly, researchers observed a similar phenotype in the angiotensin II-induce AAA model, in which genetic and pharmacological neutralization of IL-17 or use of RORγt antagonists restricted the disease (48, 49). In contrast, overexpression of suppressor of cytokine signaling (SOCS) 3 in Th17 cells reduces IL-17A and accelerates atherosclerosis. It is significant that SOCS3 has other downstream targets in addition to IL-17A, namely IL-10, which serves its own protective role in AAA (50).

#### 2.1.2 Tregs

Tregs have been reported to be detected in aortic tissue and have been shown in multiple studies to be able to play a protective role in atherosclerosis (51). As for AAA, Yodoi et al. found that the accumulation of macrophages in aneurysm tissue decreased and the number of Foxp3^+^Tregs increased, suggesting that the expansion of tregs may inhibit inflammatory cell infiltration in the blood vessel wall to prevent the formation of AAA (40). In addition, Barhoumi et al. found that Tregs play a protective role in AAA by inhibiting the infiltration of macrophages and effector T cells, accompanied by decreased plasma levels of proinflammatory cytokines IFN-γ, TNF-α and IL-6 (52). These studies suggest that through preventing the buildup of inflammatory cells and the release of pro-inflammatory chemicals, Tregs contribute to the pathophysiology of AAA. Tregs also limit AAA progression by inhibiting Cyclooxygenase-2 (COX-2) expression in bone marrow cells (53). Specifically, COX-2 is an enzyme that regulates the conversion of arachidonic acid to prostaglandins and eicosanoic acid (essential inflammatory mediators associated with the development of AAA) (54).

Further studies revealed the possible ways and mechanisms of regulating AAA by Tregs. As we all know, Coinhibitory molecule cytotoxic T-lymphocyte-associated antigen-4 (CTLA-4), a coinhibitory protein that binds to CD80 and CD86 on antigen-presenting cells and adversely controls T cell activity, is only expressed in CD4^+^ forkhead/winged-helix family of transcription factor 3 (Foxp3)^+^ Tregs (55).. Interestingly, Amin et al. found that CTLA-4 had a protective effect on Ang II-induced AAA formation in mice, which was related to the decrease in the number of efficent CD4^+^ T cells and the down-regulated expression of CD80 and CD86 co-stimulatory molecules of CTLA-4 ligand on CD11c^+^ DCs in lymphoid tissue (56). In addition, the inhibitory function of Treg cells is closely related to the acetylation level of Foxp3, which is specifically regulated by silencing information regulatory factor 1 (SIRT1) (57, 58). Jiang et al. found that EX-527, an inhibitor of SIRT1, could restore the acetylation of Foxp3 and increase the number of active Treg cells, thus restoring the inhibition ability of Treg cells to AAA (59). Notably, defective Foxp3 expression on Tregs during atherosclerotic development, resulting in switch to exTregs and up-regulation of transcription factors typical of other Th subsets, such as Th1 or Tfh (60–62). However, whether this transition will also occur in AAA has not been determined and may be a line of inquiry in the future.

Overall, further mechanistic investigations with better cell type-specific response analysis are required to clarify the relative involvement of Th1 versus Th2 versus Th17 or other CD4+ T cell subsets in contrast to other cell types producing related cytokines in AAA (63).

### 2.2 CD8^+^ T cells

Although there have been relatively few studies on the role of CD8^+^ T cells in AAA, serum of AAA patients has been found to have relatively high levels of CD8^+^ T cells compared to normal subjects. More so than patients with minor AAA, patients with large AAA had a higher level of CD8^+^ T cells (64). Early research discovered CD8^+^ CD28^+^ IFN-γ producing T cells in circulation and AAA tissue. A population of CD8+ T cells without CD27 was also found in human AAA lesions but not in peripheral blood, suggesting that this subgroup of CD8^+^ T cells may have a special function in AAA (65). Additonally, in a recent study, Zhou et al. demonstrated for the first time the pathogenicity of CD43^+^ CD8^+^ T cells in AAA by constructing a mouse AAA model induced by elastase. The study revealed that CD43 on the membrane surface of CD8^+^ T cells can induce the production of IFN-γ, which in turn participates in the inflammatory cascade, eventually promoting the development of aneurysms (65). This suggests that in addition to CD4^+^ T cells, CD8^+^ T cells can also secrete IFN-γ and participate in the regulation of AAA progression.

### 2.3 γδ T cells

γδ T cells are T cells that perform innate immune function, and their TCR consist of γ and δ chains. γδ T cells in atherosclerotic aortas were found, and it was hypothesized that they control neutrophil activation in an IL-17-dependent manner (66).. More recently, γδ2^+^ T cells were found to be more significantly in aortic aneurysm tissue compared to normal aortic tissue and peripheral blood mononuclear cell (PBMC) in AAA patients (67). Further studies showed that these γδ2^+^ T cells were the major source of IL-17A in AAA tissum, indicating that the etiology and development of AAA may be influenced by elevated IL-17A-producing γδ2^+^ T cells (67). In addition, another study by Zhang et al. revealed the specific mechanism of γδT cells in regulating the progression of AAA. The team used the porcine pancreatic elastase (PPE)-induced AAA model to reveal the pathogenicity of γδ T cells in AAA (66). Subsequently, microarray analysis found that phosphoinositide 3-kinase/AKT signaling mediated this process, providing a potential target for targeted therapy of AAA (66).

## 3 Potential therapeutic strategies targeting T cells for AAA

It is reported that homocysteine may up-regulate the expression and secretion of endogenous classifiers in endothelial cells, thereby recruiting T cells into the vascular wall and causing vascular inflammation, thus accelerating the onset of AAA (68). This indicates that lowering serum homocysteine is helpful to reduce T cell infiltration in AAA tissue, thus playing a therapeutic role. In addition, Resolvins (69), PNU-282987 (a selective α7-nAChR agonist) (70) and infliximab (TNF-α antagonist) (46) are also reported to reduce T-cell invasion in AAA, thus inhibiting the progression of AAA. These studies suggest that targeting T cells in AAA can help inhibit the progression of AAA. Therefore, it is important to find strategies to target T cells. In this section, we will discuss potential therapeutic strategies for T cells in AAA and briefly summarize the relevant drugs and mechanisms in **Table 1**.

**Table 1:** Potential therapeutic strategies targeting T cells for AAA.

Potential drugs	Targeted T cells	Mechanism	Ref.
Paricalcitol	CD4^+^T cells	Activating VDR	(71)
Dibenzazepine	CD4^+^T cells	Inhibiting Notch-γ-secretase	(72)
Doxycycline	CD8^+^T cells	/	(81)
Metformin	CD8^+^T cells	Inhibiting CD8^+^ T infiltration by lowering blood sugar	(86)
Ulinastatin	CD8^+^T cells	/	(87)
Paeonol	CD8^+^T cells	Inhibiting the NF-κB pathway	(90)
Interleukin	Tregs	/	(77)
SCFAs	Tregs	/	(80)

### 3.1 Targeting CD4^+^ T cells

In the previous section, we have summarized the mechanisms by which various subgroups of CD4^+^ T cells participate in regulating the formation of AAA. Therefore, drugs targeting CD4^+^ T cells may act to inhibit the progression of AAA. Since the vitamin D receptor (VDR) has been demonstrated to have potent immunomodulatory properties *in vitro* and *in vivo* studies, researchers are interested in exploring the role of activating VDR-agonists in regulating the progression of AAA (71). Nieuwland et al. collected aneurysm wall samples during surgery and studied inflammatory footprints. It was found that a brief intervention with paricalcitol (VDR-agonists) resulted in a 73% selective reduction in CD4^+^ T helper cells, demonstrating that the VDR agonist paticalcitol significantly lowers local inflammation by inhibiting the calcineurin/NFAT signaling cascade and CD4+T cell activation (71). In another study, the Notch γ-secretase inhibitor dibenzazepine (DBZ) clearly blocked Ang II-stimulated macrophage and CD4^+^ T cell accumulation in an Ang II-induced mouse model, simultaneously reversed Th2 response *in vivo* through Notch signaling, demonstrating the potential for DBZ as a new therapeutic agent for the treatment of AAA (72). Unfortunately, these drugs are still in the preclinical stage and have not yet entered clinical trials. Further clinical trials are needed to verify their efficacy in the future.

To date, drugs targeting Tregs to inhibit the progression of AAA are still in preclinical studies. IL is a lymphoid factor that interacts between white blood cells and immune cells. It is crucial for information transmission, for engaging and controlling immunological responses, for influencing T and B cell activation, proliferation, and differentiation, and for playing a significant part in inflammation (73). Particularly, endothelial and epithelial cells at barrier sites express the IL-1 family member IL-33 constitutively, and its expression is stimulated in inflammatory cells infiltrating the inflammatory site (74–76). Li et al. used C57BL/6J mice to construct the AAA model. The results confirmed IL-33 suppresses AAA by enhancing Tregs expansion and activity, indicating that regimens that reprogram IL-33 or increase endogenous IL-33 expression may limit the progression of mature AAA or prevent the development of human AAA (77). However, although recombinant IL-19 was shown to inhibit the formation and progression of experimental AAA, it did not affect the invasion level of Tregs (78). This indicates that the mechanism of intervention of different interleukins in aortic aneurysm is quite different, and not all interleukins can inhibit AAA by promoting the amplification of Tregs, so the search for Tregs with immunomodulatory effects will be the direction of future exploration. Short-chain fatty acids (SCFAs), a metabolite produced by intestinal microbes, have been shown to enhance the number of Treg cells in the colonic lamina propria (cLP) and to protect against non-intestinal inflammatory disorders such atherosclerosis and post-infarction cardiac inflammation (79). Propionic acid protects against AAA, according to Yang et al., by encouraging the recirculation of cLP-Tregs through colonic draining lymph nodes to inflamed aorta (80). These findings demonstrate the crucial part SCFAs play in aortic inflammation and lay the groundwork for the creation and application of prebiotic-based therapies for human AAA.

### 3.2 Targeting CD8^+^ T cells

Regarding CD8+ T cells, a prior clinical trial by Lindeman et al. randomly assigned 60 patients to receive either no treatment (control group) or two weeks of low-, medium-, or high-dose doxycycline (50, 100, or 300 mg/d, respectively). Following that, samples of the aorta wall were taken during surgery, and the impact of doxycycline treatment on vascular inflammation was assessed (81). Doxycycline has an inhibitory effect on AAA, which is usually attributed to the inhibition of MMP-9. However, this study shows that Doxycycline can still selectively reduce the content of 95% CD8+ T cells in aortic wall through activating protein-1 (AP-1) (81). The findings of this study have implications for the stabilization of abdominal aneurysms by medication and perhaps for other inflammatory diseases involving CD8^+^ T cells. In addition, epidemiological evidence has shown that diabetics are less likely to develop AAAs, and when AAAs are present, the progression or expansion of AAAs is slower in diabetics (82–85). Based on this evidence, researchers explored the effect of metformin on the progression of AAA. This clinical study included 58 patients, and in experimental modeling it was found that metformin significantly inhibited the formation and progression of AAA, and reduced the infiltration of aortic mural macrophages and CD8^+^ T cells (86). However, the specific pathway of Metformin affecting AAA is still unknown, and its effect on CD8^+^ T cells seems to lack specificity. In addition to the above clinical studies, preclinical studies conducted in recent years have also identified drugs that target CD8^+^ T cells to inhibit the progression of AAA. For example, Ulinastatin inhibited CD8^+^ T cells in the aortic wall of AAA mice induced by PPE and limited the formation and progression of experimental AAA (87). However, this study did not directly prove the effect of CD8^+^ T cells on AAA through depletion of CD8^+^ T cells or other interventions. The solution could be to construct AAA models by depleting CD8^+^ T cells mouse and then compare the limiting effect of ulinastatin on AAA. Paeonol, which has been shown to have anti-inflammatory and cardiovascular protective characteristics, was used in another investigation (88, 89), was shown to block the progression of experimental AAA by inhibiting the NF-κB pathway, and the infiltration of CD68^+^ macrophages and CD8^+^ T cells was significantly reduced when paeonol was taken together (90). There are not enough experiments to prove that Paeonol has a specific inhibitory effect on CD8^+^ T cells on the AAA vascular wall. This study has the same limitations as the above studies and may be the direction of future research.

## 4 Conclusion and prospects

In the past ten years, it has become clear that immune cells play a role in the pathogenesis of AAA, and specific immune cells have been found in AAA lesions. Numerous mechanistic investigations have offered proof of the part immune cells play in the etiology of AAA. Given the unique role of T lymphocytes, additional research utilizing cell type-specific knockouts and more physiologically accurate models is required, which is still poorly known. This article summarizes the animal and clinical evidence from various T cell subsets in AAA, and summarizes potential therapeutic strategies targeting T cell subsets. Existing evidence supports that CD4^+^ T cells and CD8^+^ T cells play a pro-inflammatory role in AAA, thus promoting the formation and progression of AAA, while the amplification of Tregs restricts AAA. Several drugs targeting CD8^+^ T cells have been carried out clinical studies and shown certain efficacy. However, drugs targeting CD4^+^ T cells and Tregs are still in the pre-clinical research stage and need further clinical verification. At the same time, the targeted therapy for T cells in AAA also has certain limitations and challenges. For example, although Tregs amplification limits the progress of AAA, cell-based therapy has high costs, difficulties in production or the need for special equipment, which leads to difficulties in clinical transformation. On the other hand, the dosage and pharmacokinetics of cell therapy are also difficult to determine, and there are differences in the survival and proliferation of cells within different individuals, which may lead to differences in their efficacy. How to target and deliver drugs targeting T cells to the vascular wall of AAA is also an urgent problem to be solved.

Animal models of AAA formation have been created and are frequently utilized in experimental research, however they do not accurately represent human pathophysiology. Many therapeutic drugs have achieved great success in animals, but are not effective or even counterproductive in clinical practice. There are many reasons for this. For example, unlike humans, mice often develop suprrenal AAA. The three classical animal models are still insufficient to accurately simulate the formation process of chronic AAA in humans, and the cellular and molecular mechanisms are different *in vivo* and *in vitro*. The ideal of better bioavailability and fewer side effects is still a long way off. Therefore, how to select suitable and possibly effective targets from the inflammatory network is still one of the major challenges. The role and mechanism of novel anti-inflammatory factors and T cells in AAA remain to be clarified, and the new mechanism may bring new targets. A potential new field called “macrophage phenotype polarization” may help find important AAA-related regulators of chronic inflammation. It is being investigated if macrophages in AAA tissue have a stronger M1 or M2 phenotype and how to change the M1/M2 balance. In addition to changes in inflammation of AAA blood vessel walls, the imbalance between protective factors and pro-inflammatory molecules in perivascular adipose tissue may also lead to vascular dysfunction. In the future, anti-inflammatory therapy is expected to be a game-breaker for AAA prevention and treatment.

## Author contributions

WG wrote the manuscript and created the figures. A comprehensive collection and preparation of related papers was undertaken by YT, while LL conceived and approved the final version to be submitted. All authors contributed to the article and approved the submitted version.
